# Nanoscale Iron-Based Metal–Organic Frameworks: Incorporation of Functionalized Drugs and Degradation in Biological Media

**DOI:** 10.3390/ijms24043362

**Published:** 2023-02-08

**Authors:** Ioanna Christodoulou, Pengbo Lyu, Carla Vieira Soares, Gilles Patriarche, Christian Serre, Guillaume Maurin, Ruxandra Gref

**Affiliations:** 1 Institut de Sciences Moléculaires d’Orsay, UMR CNRS 8214, Université Paris-Sud, Université Paris-Saclay, 91400 Orsay, France; 2 Institut Charles Gerhardt Montpellier, UMR 5253 CNRS, UM, ENSCM, University of Montpellier, 34293 Montpellier, France; 3 Center for Nanoscience and Nanotechnology, C2N UMR 9001, CNRS, Université Paris Sud, Université Paris Saclay, 911128 Palaiseau, France; 4 Institut des Matériaux Poreux de Paris, ENS, ESPCI, CNRS, PSL University, 75000 Paris, France

**Keywords:** metal–organic frameworks, drug loading, stability, biological media, prednisolone, STEM-HAADF, biodegradable nanoparticle

## Abstract

Metal–organic frameworks (MOFs) attract growing interest in biomedical applications. Among thousands of MOF structures, the mesoporous iron(III) carboxylate MIL-100(Fe) (MIL stands for the Materials of Lavoisier Institute) is among the most studied MOF nanocarrier, owing to its high porosity, biodegradability, and lack of toxicity. Nanosized MIL-100(Fe) particles (nanoMOFs) readily coordinate with drugs leading to unprecedented payloads and controlled release. Here, we show how the functional groups of the challenging anticancer drug prednisolone influence their interactions with the nanoMOFs and their release in various media. Molecular modeling enabled predicting the strength of interactions between prednisolone-bearing or not phosphate or sulfate moieties (PP and PS, respectively) and the oxo-trimer of MIL-100(Fe) as well as understanding the pore filling of MIL-100(Fe). Noticeably, PP showed the strongest interactions (drug loading up to 30 wt %, encapsulation efficiency > 98%) and slowed down the nanoMOFs’ degradation in simulated body fluid. This drug was shown to bind to the iron Lewis acid sites and was not displaced by other ions in the suspension media. On the contrary, PS was entrapped with lower efficiencies and was easily displaced by phosphates in the release media. Noticeably, the nanoMOFs maintained their size and faceted structures after drug loading and even after degradation in blood or serum after losing almost the totality of the constitutive trimesate ligands. Scanning electron microscopy with high annular dark field (STEM-HAADF) in conjunction with X-Ray energy-dispersive spectrometry (XEDS) was a powerful tool enabling the unraveling of the main elements to gain insights on the MOF structural evolution after drug loading and/or upon degradation.

## 1. Introduction

Nanomedicine provides the opportunity to organize matter at the nanometer scale, creating drug reservoirs with a particular interest in treating severe diseases, such as cancer and infections. Drug nanocarriers efficiently incorporate, protect against degradation, and ferry the active molecules from the administration site to their target, improving their pharmacokinetics [1]. A variety of natural and synthetic materials have been engineered at a nanoscopic level and explored for drug delivery. Liposomes, the first nanotechnology-based drug delivery system, were discovered early in the 1960s [2], and since then, many other types of drug nanocarriers have been developed, including the organic or inorganic nanoparticles (NPs): polymeric and metal NPs, quantum dots, carbon nanotubes, micelles, nanogels, and dendrimers. Later, a report described the first application of hybrid organic-inorganic NPs for drug delivery applications [3].

Among them, the nanoscale mesoporous iron trimesate metal–organic framework MIL-100(Fe) or nanoMOFs (MIL stands for the Material of Institute Lavoisier) attracts a growing interest due to its potential in the biomedical field [3,4]. It can incorporate high payloads of a large variety of active molecules thanks to its highly porous structure and intrinsic amphiphilic microenvironment [5,6,7,8,9]. Additionally, these nanoMOFs were shown to be biodegradable and poorly toxic both in vitro and in vivo at high doses without any adverse side effects, even after repetitive administrations [10,11,12,13,14,15,16,17]. When nanoMOFs were in contact with media containing phosphate ions, rapid degradation was observed due to the progressive replacement (within a few hours) of the constitutive trimesate ligands by phosphates [18]. NanoMOFs presented the highest stabilities in acidic pH in gastric and simulated intestinal media [19]. Finally, it was shown that these nanoMOFs were less degraded in serum than in phosphate-buffered saline (PBS), possibly because of the formation of a protective protein corona [10,19]. NanoMOFs were effective in avoiding cancer metastases when administered in an animal model of mice bearing lung tumors [15]. The supposed mechanism was associated with a rapid aggregation of the nanoMOFs in the lung capillaries, followed by drug release and disaggregation due to degradation. 

In vitro studies showed that contrary to purely organic or inorganic NPs previously mentioned, these hybrid nanoparticles interact with their surrounding media by continuously changing their composition. For instance, phosphates from PBS coordinate with iron sites and eventually erode the nanoMOFs leading to the formation of an inorganic skeleton [18,20]. However, the in vivo degradation mechanism of MIL-100(Fe) nanoMOFs is still not yet fully understood. Blood contains more than two thousand proteins and a variety of ions and other molecules, including phosphates. The erosion mechanism and the composition of nanoMOFs in such a complex media are still unraveled, and this is one main aim of this study. To do so, firstly, the interaction of nanoMOFs with single ions (phosphates and sulfates) was investigated. Density Functional Theory (DFT) calculations were performed on representative clusters of the MOF to assess the strength of these ions/MOF interactions, whereas STEM-HAADF was used to image the nanoMOFs with atomic precision without damaging them before and after their degradation in various media, including blood and serum. Of main interest, STEM-HAADF coupled with energy-dispersive X-Ray analysis (XEDS) provided valuable information about the elements which were irreversibly associated with the nanoMOFs after their incubation in the studied media. Compared with other nanoplatforms (polymeric or lipid nanoparticles, micelles, liposomes, dendrimers) nanoMOFs are porous and thus require additional characterization methods, such as porosity and crystallinity investigations.

Similarly, some of us have shown that nanoMOFs interact with drugs that coordinate with their iron sites leading, in some cases, to strong binding and almost perfect encapsulation efficiencies (EE). For instance, phosphorylated antiviral and anticancer drugs were successfully incorporated by soaking from aqueous solutions reaching loadings up to 35 wt % [5,21,22]. Agostoni et al. characterized these interactions by successfully entrapping mono- and tri-phosphorylated derivatives of azidothymidine (AZT-MP and AZT-TP, respectively) into MIL-100(Fe) nanoMOFs and investigated their release kinetics under simulated physiological media [22]. The monophosphorylated AZT was released faster as compared with triphosphorylated AZT due to its weaker interactions with the unsaturated iron(III) Lewis acid sites of the framework. In a further study, it was shown that the nanoMOFs loaded with AZT-TP presented higher stability against degradation as compared with the ones loaded with AZT-MP [23]. The anticancer drug gemcitabine monophosphate was encapsulated, reaching a maximal loading of 30 wt % with an excellent encapsulation efficiency (>98%), validating once again the good affinity of phosphates for the iron sites [5].

These studies suggest that the nanoMOFs’ stability depends on the composition of the media they are in contact with and also on the presence of active molecules in their porosity. However, the above-mentioned reports focused only on nanoMOFs loaded with drugs bearing phosphate functional groups. To gain further insights into the interplay between the interactions between drug–nanoMOFs and their external media, in this study, a family of glucocorticoids (GCs) bearing or not bearing functional groups (phosphates and sulfates) was used. Indeed, both phosphate and sulfate moieties are prone to coordinate iron metal sites. GCs are synthetic analogs of natural steroid hormones presenting anti-inflammatory and immunosuppressive activity [24]. In particular, prednisolone has been extensively studied for the treatment of autoimmune diseases, such as rheumatoid arthritis [25], allergies, and asthma [26], as well as in cancer therapy to increase the effectiveness of chemotherapy and/or to reduce side effects [27]. However, the systemic and long-term use of prednisolone is hampered by severe side effects, low bioavailability, and an off-target biodistribution profile. To overcome these limitations, ‘’smart’ nanocarriers able to encapsulate GCs were developed to improve the therapeutic efficiency of prednisolone and to achieve a sustained and controlled release.

PP is widely used for ocular anti-inflammatory therapy, and several formulations have already been approved by US Food and Drug Administration (FDA) [28]. In this study, prednisolone and its phosphorylated and sulfated analogs (PP and PS) were incorporated for the first time in MIL-100(Fe) nanoMOFs. Monte Carlo simulations enabled describing the filling of the cages by the drugs, while DFT calculations were carried out to shed light on the affinities of prednisolone and its functionalized analogs of PP and PS forms with MIL-100(Fe). Complementary physicochemical methods were employed for an in-depth characterization of the drug-loaded nanocarriers. These techniques allowed for following any changes in the loaded nanoMOFs, including size and morphology, as well as their stability and release studies [29]. The drug-loaded nanoMOFs were allowed to degrade in media containing phosphates and/or sulfates to analyze the competitive interactions between the incorporated drugs and the nanoMOFs’ surrounding media. In parallel, the release kinetics of the trimesate ligand was evaluated for a better comprehension of the nanoMOFs’ stability once loaded with the drugs in different media. Moreover, we systematically explored the morphological and chemical stability of empty and loaded MIL-100(Fe) nanoMOFs in different media using STEM-HAADF coupled with XEDS.

The physicochemical properties of nanocarriers, such as their size, surface charge, shape, etc., play a key role in their efficacy and in vivo fate. Different degradation pathways have been reported depending on the functional material and its properties. Such pathways include hydrolysis, oxidative degradation, enzymatic degradation, and so forth. A series of methods was applied to assess the degradation mechanism of any functional material. These methods comprise analytical and spectroscopic techniques, microscopic observations, theoretical simulations, and many more [30]. In this study, morphological changes of empty nanoMOFs and elements distribution upon incubation in serum and blood were observed by STEM-HAADF. To the best of our knowledge, there are no studies analyzing the morphology and composition of nanoMOFs upon incubation in such media. Such investigations are crucial for a deeper understanding of the degradation mechanism in conditions closer to these of the living organism.

## 2. Results and Discussion

### 2.1. Synthesis and Characterization

**Figure 1:**
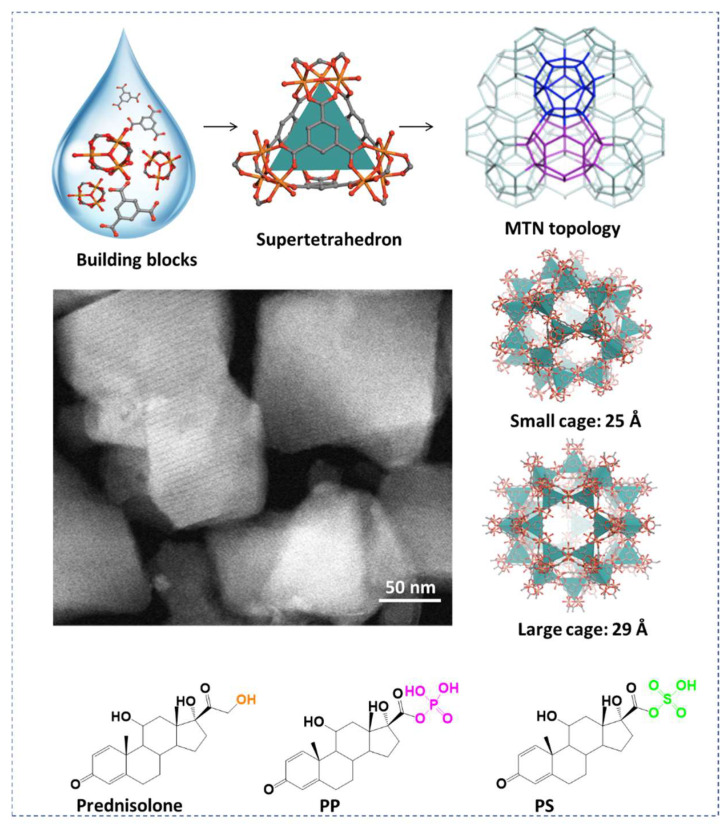
Schematic representation of MIL-100(Fe)’s structure resulting from iron(III) trimers and trimesic acid assembly. The three active molecules, prednisolone, PP, and PS, were encapsulated into the nanoMOFs’ large cages by overnight impregnation.

MIL-100(Fe) is built up from the self-assembly of iron(III) trimers linked with organic trimesate ligands, forming super tetrahedra, which further assemble, leading to a 3D micro/mesoporous structure of MTN topology [31]. The resulting structure consists of small and large cages with diameters of around 25 Å and 29 Å, respectively, and they are accessible through microporous pentagonal (5.6 Å) and hexagonal (8.6 Å) windows, respectively [32,33]. A ‘’green’’ microwave-assisted hydrothermal method was used here to obtain submicronic particles of controlled sizes (nanoMOFs), as has been previously described [7,18]. The crystallinity of the nanoMOF was confirmed from their diffraction patterns (Figure S1), in line with previous studies [34,35]. STEM-HAADF electron microscopy investigations revealed that the nanoMOFs possessed faceted structures (Figure 1). The nanoMOFs’ average hydrodynamic diameter was 230 ± 20 nm, according to DLS investigations, with a polydispersity index (PdI) of 0.09, indicating their homogeneous distribution. 

The three drugs, prednisolone, PP, and PS, were successfully loaded within the nanoMOFs’ internal porosity by overnight incubation from aqueous solutions (Figure 1). The synthesized nanoMOFs acted similarly to molecular sponges soaking the drugs in their internal porosity. The molecular dimensions of the flexible drugs, prednisolone (14.2 × 8.5 and 7.5 Å), PP (17.8 × 9.5 and 7.3 Å), and PS (17.2 × 8.4 and 8.0 Å), are compatible only with the large hexagonal windows (4.7 × 5.6 and 8.6 Å) of the rigid nanoMOFs, and not with the pentagonal ones (Table S1).

The optimized drug loading and encapsulation efficiencies (EE) for prednisolone, PS, and PP were determined by HPLC. PP, the phosphorylated form of prednisolone, presented both the highest drug loadings (30 wt %) and EE (~95%) as compared with the other two drugs (19 and 57 wt % for prednisolone and 15 and 72% for PS, respectively). The almost perfect EE for PP is in line with previously reported studies, showing that phosphate drugs, such as gemcitabine-phosphate and AZT-phosphate, were entrapped with EE close to 100% [5,22,36].

**Figure 2:**
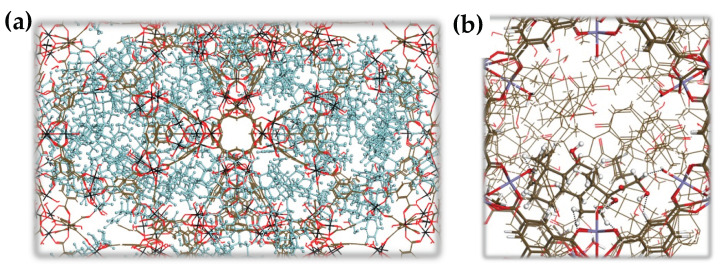
Pore filling of prednisolone in MIL-100(Fe) revealed by Monte Carlo simulations. The characteristic guest/MOF interacting distances are indicated by the black-dashed line. Color code: C (dark gray), O (red), H (white), and Fe (black). The guest molecules are represented by the light cyan color in (**a**,**b**) to clarify the visualization.

Figure 2a,b illustrate the filling of the nanoMOF cages by prednisolone at saturation. One can see that prednisolone is preferentially distributed in the vicinity of the inorganic node in the windows of the large cages (Figure 2b). The host/guest separating distances of about 3 Å evidence the presence of van der Waals-type interactions, with the hydroxyl functions of prednisolone pointing towards the oxo-trimers.

**Figure 3:**
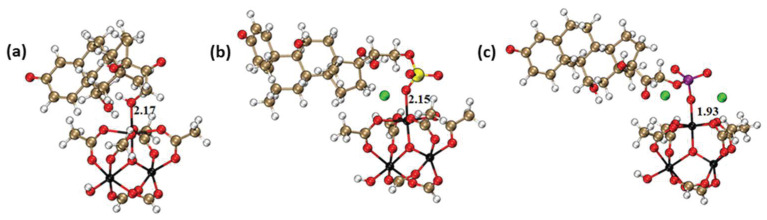
DFT-optimized most stable drug@Fe-CUS loading configurations for (**a**) prednisolone, (**b**) PP, and (**c**) PS. Color code: C (brown), O (red), H (white), ion (black), S (yellow), P (purple), and Na (green). The distances are in Å.

Prednisolone loading of 19 wt % was achieved after overnight impregnation with the nanoMOFs. However, the EE was only 57%, suggesting that this molecule does not exhibit strong interactions with the nanoMOF framework. In contrast, PS showed a significantly higher EE of 72%, which could possibly be attributed to stronger interactions with the MOF framework. These observations can be understood in light of the DFT calculations performed using a cluster representative of the oxo-trimer of MIL-100(Fe). These calculations enabled us to determine the most stable adsorption configurations of the three drugs and their affinity towards the Fe-oxo-trimer. Figure 3 shows that the three drugs bind to the coordinatively unsaturated sites (CUS) via the oxygen atoms of their functional groups, i.e., -OH, -OSO_3,_ and -OPO_3_ associated with characteristic Fe-O distances of 2.17 Å, 2.15 Å, and 1.93 Å, respectively (for comparison, the Fe-O distance is 2.05 Å in the case of Fe CUS coordinated by water) [37]. The corresponding interaction energies were found to be −156 kJ.mol*^−^*^1^, −228 kJ.mol*^−^*^1,^ and −289 kJ.mol*^−^*^1^ for prednisolone, PS, and PP, respectively. Therefore, the Fe-oxo-trimer affinity to the drugs is predicted to increase in the order of prednisolone < PS < PP, consistent with the experimental trend observed when incorporating these drugs in the nanoMOFs.

**Figure 4:**
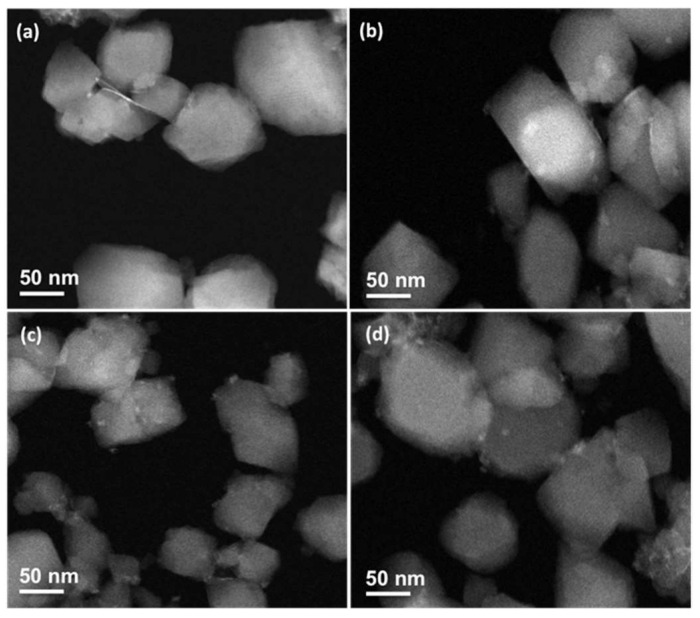
STEM-HAADF images of nanoMOFs before (**a**) and after loading with 19 wt % prednisolone (**b**), 30 wt % PP (**c**), and 15 wt % PS (**d**).

STEM-HAADF microscopy was performed to observe morphological changes after prednisolone, PS, and PP loading. Whatever the loaded drug, the nanoMOFs maintained their faceted morphology (Figure 4b–d), and their average size measured on more than 100 individual particles was similar (prednisolone, 155 ± 57 nm; PS, 147± 30 nm; and PP, 161 ± 58 nm) as compared with the empty nanoMOFs (141 ± 37 nm).

PXRD patterns of nanoMOFs before and after loading confirmed the maintenance of their crystalline structure in the presence of prednisolone, PP, and PS (Figure S2). The three incorporated drugs were crystalline (Figure S2), displaying their main characteristic Bragg peaks. These peaks were undetectable in all drug-loaded nanoMOFs, suggesting the absence of residual-free crystalline drug molecules. This clearly shows that all the drugs were incorporated in a molecular state inside the cages.

Infrared spectroscopy investigations pointed out the interactions between the drugs and the framework after their impregnation (Figure S3). More precisely, the bands at 1700 cm*^−^*^1^, corresponding to the v(C=O) stretching of prednisolone, PP, and PS were slightly shifted once encapsulated into the nanoMOFs [38,39]. These results are in line with previous studies showing similar shifts in the case of acetylsalicylic acid and caffeine incorporation in MIL-100(Fe) nanoMOFs [40,41].

STEM-HAADF microscopy coupled with XEDS is a powerful tool to shed light on the distribution of the incorporated drugs after loading. Among the three drugs, only PS and PP have distinctive elements (P and S) different from the nanoMOFs containing solely O, C, and Fe atoms. NanoMOFs contain large amounts of coordinated water molecules, which cannot be removed, even under a high vacuum (in our conditions). Therefore, information on the O distribution obtained by STEM-HAADF–XEDS arises both from coordinated water, drug molecules, and trimesate ligands. Moreover, the sample holders contain C, making it difficult to separate information arising from C atoms in the grids and the analyzed samples. In conclusion, we based our investigations on Fe atoms, which were shown to remain associated with the nanoMOFs without being released in the degradation media [18].

**Figure 5:**
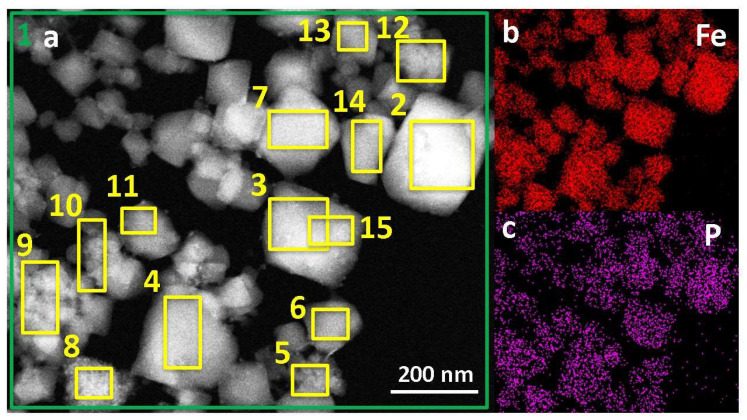
(**a**) Typical STEM-HAADF images of nanoMOFs loaded with PP at a DL of 30 wt %. (**b**,**c**) Elemental distribution of Fe (red) and P (violet) into the nanoMOFs after PP loading. Yellow rectangles represent the selected regions of interest, and the green rectangle corresponds to the quantification of the elements in the entire image. The scale bar represents 200 nm.

Figure 5 presents the elemental distribution of Fe (representative of the MOF iron sites) and P (representative of PP) after the encapsulation of PP into the nanoMOFs. Globally, PP appears homogeneously distributed inside the particles (Figure 5b). Additionally, the P/Fe ratio was calculated for different regions of interest (indicated by yellow rectangles in Figure 5a) to confirm the samples’ homogeneity further. The P/Fe ratio corresponds to the P atoms (or drug molecule) bound per each iron site of the nanoMOFs (Fe normalized at % equals 2.0 ± 0.3 and P normalized at % to 0.3 ± 0.1). Noteworthy, the P/Fe average values experimentally found by elemental analysis (0.5 ± 0.1) (Table S2) are in good agreement with the theoretical calculations (0.6), representative of the occupancy of the available iron sites from PP, after taking into account the experimental drug loading determined by HPLC (30 wt %) (for details, see theoretical calculations in SI).

These results show a homogeneous distribution of PP in the nanoMOFs and a good agreement between individual STEM-HAADF–XEDS investigations and the data obtained by HPLC. In the case of PS, a similar homogeneous drug distribution within the nanoMOFs was found (Figure S4), as revealed by the constitutive S-element distribution. However, more precise quantifications were not possible, mainly because, contrary to PP, the PS molecules were prone to be released out of the nanoMOFs during the sample preparation (washings and drying) necessary for microscopy investigations. Presumably, this was due to PS and prednisolone’s much weaker interactions with the framework, as compared with PP.

### 2.2. Degradation Study of Empty nanoMOFs

**Figure 6:**
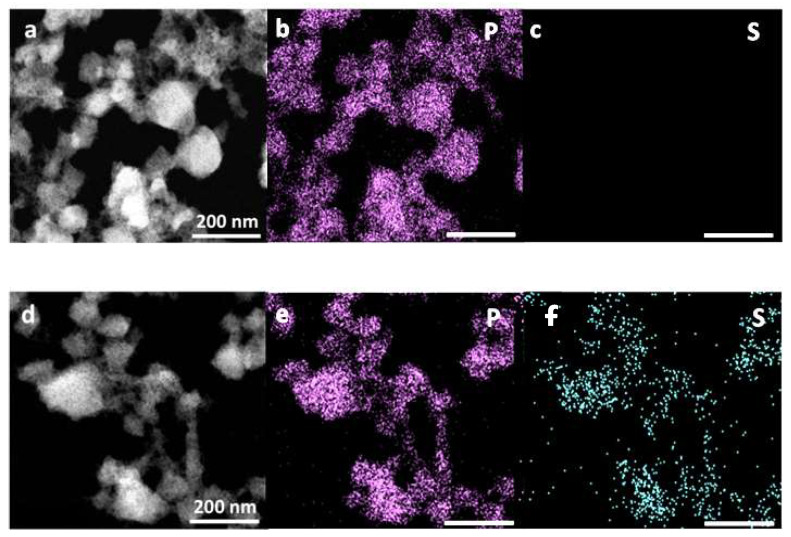
(**a**,**d**) Typical STEM-HAADF images of nanoMOFs (0.25 mg mL*^−^*^1^) after 48 h incubation in PBS and a media containing equimolar amounts of Na_2_SO_4_ and Na_2_HPO_4_. (**b**,**c**,**e**,**f**) STEM–XEDS elemental analysis of degraded nanoMOFs. P (purple) and S (blue).

High-resolution STEM-HAADF coupled with XEDS was further used to investigate the morphological and chemical composition changes of the nanoMOFs upon degradation in the presence of phosphates contained in PBS. After two days of incubation, the structure became amorphous and spongious, but remarkably the nanoMOFs did not collapse (Figure 6a). During the same incubation time, the amount of released trimesate constitutive ligand was 98 ± 2%, suggesting a total degradation. The P/Fe ratio was found to be 0.7 ± 0.1, suggesting that around two phosphate molecules were bound per each iron trimer.

This amount is higher than the P/Fe ratio obtained in the case of PP loading (0.5 ± 0.1) and could be explained by the fact that PP molecules can only access large cages, whereas the phosphate ones can access both small and large cages. These data support the high affinity of phosphate molecules for the iron sites inside the nanoMOFs. 

In a complementary study, nanoMOFs were incubated in a medium containing both sulfate and phosphate ions in an equimolar ratio to investigate their competition for the iron sites. Figure 6c shows that after incubation, the nanoMOFs presented similar morphologies as those incubated in phosphate media (Figure 6d). They incorporated both P from phosphates and S from sulfates, and these elements were colocalized with Fe, showing that they were incorporated inside the nanoMOF matrix. However, the S amounts were close to the detection limit (<0.1%) as compared to P amounts, which were 0.7 + 0.1, similar to the case of nanoMOF incubation in PBS.

These findings highlight the preferential affinity of the nanoMOFs for phosphates as compared to sulfates. This result is in agreement with the complexation constants of Fe (III) with the phosphates and sulfates already reported [42]. It was, therefore, interesting to study the release of the drugs bearing phosphate and sulfate moieties in media containing phosphates or sulfates.

### 2.3. Comparative Study of Release Kinetics in Different Media

The release of the three drugs and nanoMOF degradation was first studied in PBS (Figure S5). Even after one month of incubation in PBS at 37 °C, prednisolone release did not exceed 60% of the entrapped amount, possibly due to its poor solubility in aqueous media. Indeed, as compared with PP and PS, which are freely soluble in aqueous media, prednisolone is a hydrophobic molecule with a solubility in water of only around 2 10^−2^ mg L^−1^ (Table S3). It was necessary to add ethanol, a good solvent for the drug, to the release medium to release more prednisolone from the nanoMOFs. In this way, prednisolone release reached 75% within 24 h, as measured by HPLC.

Prednisolone and PS were released in a “burst manner”, with 53% and 87% in the first 90 min after incubation and after 6 h, respectively. On the contrary, PP was released in a controlled manner, reaching 40% after 3 h and 100% after 48 h of incubation. This suggests a lower affinity of prednisolone and PS for the nanoMOFs as compared with PP, in agreement with molecular simulations. The sustained release of PP was also in line with previous studies on the release of phosphate molecules, such as AZT-TP from MIL-100(Fe) nanoMOFs [21].

In parallel, the release of the constitutive ligand (trimesate) was also quantified to gain insights into the possible influence of the encapsulated molecules on the nanoMOFs’ stability. Figure S5b summarizes the kinetics of ligand release from the nanoMOFs loaded or not loaded with drugs. Empty nanoMOFs lost 70% of their constitutive ligand within 30 min and totality within 3 h. This is in agreement with the rapid erosion of the nanoMOFs in PBS. The erosion was slightly reduced in the case of nanoMOFs loaded with prednisolone (60%) and PS (66%) at the same time point (30 min). Interestingly, the trimesate ligand release was drastically reduced for PP nanoMOFs loaded with PP (23% after 30 min and 38% after 3 h), suggesting that the firm binding of PP to the nanoMOFs’ iron site that stabilizes their structure from phosphate degradation.

**Figure 7:**
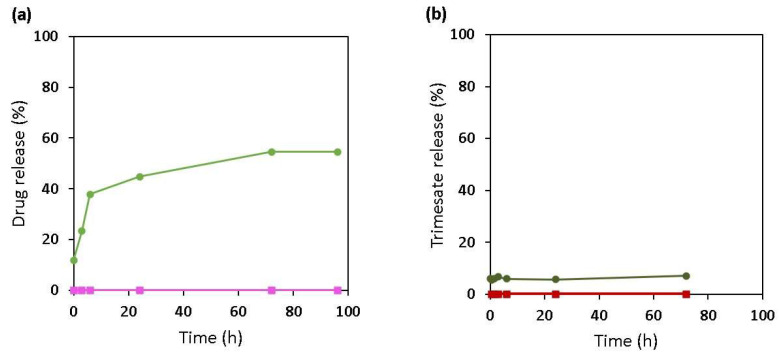
(**a**) Comparison of PP (pink) and PS (green) release in sulfates. (**b**) Trimesate loss of nanoMOFs with PS (DL = 15 wt %) (dark green) and PP (DL = 30 wt %) (dark red) in sulfate-containing media, pH 7.4, 37 °C. The sample concentration was 0.5 mg mL^−1^.

Furthermore, drug and ligand release was evaluated in a medium containing sulfate ions instead of phosphate ones (Figure 7) in an attempt to gain more insights into the competition between the ions of the degrading solution and the functional groups of the drugs. Of note, prednisolone was not selected for this study due to its poor solubility in aqueous media, as previously indicated. Interestingly, PP was not released even after 3 days of incubation in sulfate media, in contrast with PS, which reached around 50% (Figure 7a). This suggests that sulfate ions from the release media diffuse inside the nanoMOFs and replace the coordinated PS, similarly to phosphate ions from the media replacing PP and leading to its release.

A low trimesate release (<10%) was observed for both PP- and PS-loaded nanoMOFs. These results clearly show the stability of the nanoMOFs in sulfate media as compared with phosphate ones (Figure 7b), once again due to the lower binding affinity of sulfates towards iron(III) in comparison with phosphates. In a nutshell, the release and degradation studies are both in agreement with DFT calculations, showing that phosphates (from the media or linked to the drugs) have a higher affinity towards the representative cluster of the MOF than sulfates, as shown in Figure 3.

The studies performed in media containing only a single type of ions (phosphates or sulfates) were a valuable tool to explore the morphological and compositional changes of the particles. However, it is important to study the stability of the nanoMOFs in more complex media close to the living organism to explore the effect of different ions on the degradation mechanism of the MOFs matrices. Therefore, bare nanoMOFs were incubated in serum and blood, and their modifications were monitored by STEM-HAADF–XEDS.

### 2.4. Degradation Study in Blood and Serum

MIL-100(Fe) nanoMOFs were administered by an intravenous route, and it was found that they released their constitutive trimesate ligands, but to the best of our knowledge, there is no information about the nanoMOF composition once in contact with a complex media such as blood. Blood is composed of various cells suspended in plasma, which constitutes around 55% of blood fluid and is composed mostly of water (92 vol %), more than two thousand proteins, glucose, mineral ions, hormones, carbon dioxide, and other molecules. Albumin is the main protein in plasma, whereas mineral ions comprise Na^+^, K^+^, Mg^2+^, Ca^2+^, carbonates, phosphates, and sulfates [43,44].

The previous results obtained in the competition studies between phosphates and sulfates point out the major role of phosphates in drug entrapment as well as nanoMOF degradation. More complex competitions are expected to occur when the nanoMOFs interact with blood. Thus, it was interesting to investigate further, using our methodology based on STEM-HAADF–XEDS microscopy, the nanoMOFs’ composition after contact with whole blood and serum. The nanoMOFs were incubated in these media at a concentration of 0.5 mg mL^−1^, corresponding to the administered dose of nanoMOFs in rats used in previous in vivo studies [13,45]. Two different time points were chosen (2 h and 48 h) to account for the early and late fate of the nanoMOFs.

**Figure 8:**
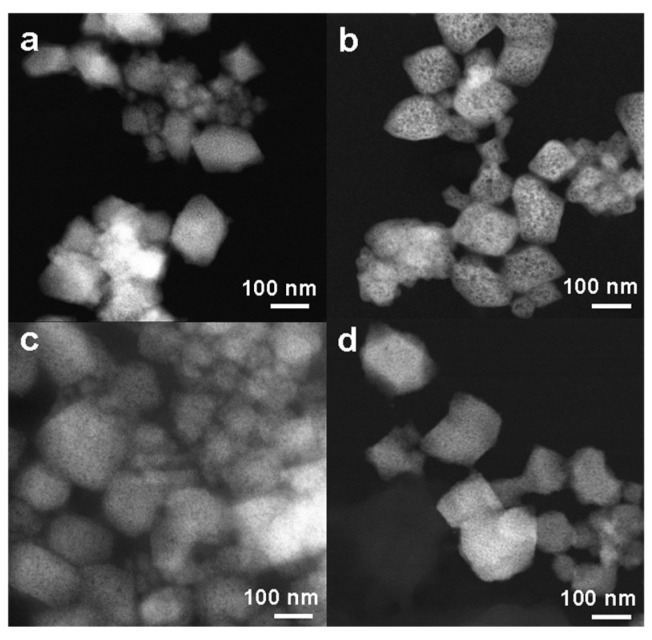
STEM-HAADF images of nanoMOFs after incubation in (**a**,**b**) serum and blood for 2 h and (**c**,**d**) serum and blood for 48 h. The sample concentration was 0.5 mg mL^−1^.

Figure 8 presents the morphology of the nanoMOFs recovered from these media after washing with Milli-Q water to remove the proteins and other compounds not firmly bound. Noteworthy, the nanoMOFs maintained their faceted structure with holes visible, especially in blood, after 2 h (Figure 8b). These holes present a similarity with those observed during nanoMOFs’ degradation in PBS (Figure 6a). Remarkably, the nanoMOFs’ average particle sizes were maintained even after 2 days of incubation in both media, as indicated by the slight variation observed after evaluating more than 100 nanoparticles (141 ± 37 nm for (a) 145 ± 30 nm, (b) 160 ± 34 nm, (c) 160 ± 40 nm and (d) 144 ± 25 nm). 

**Figure 9:**
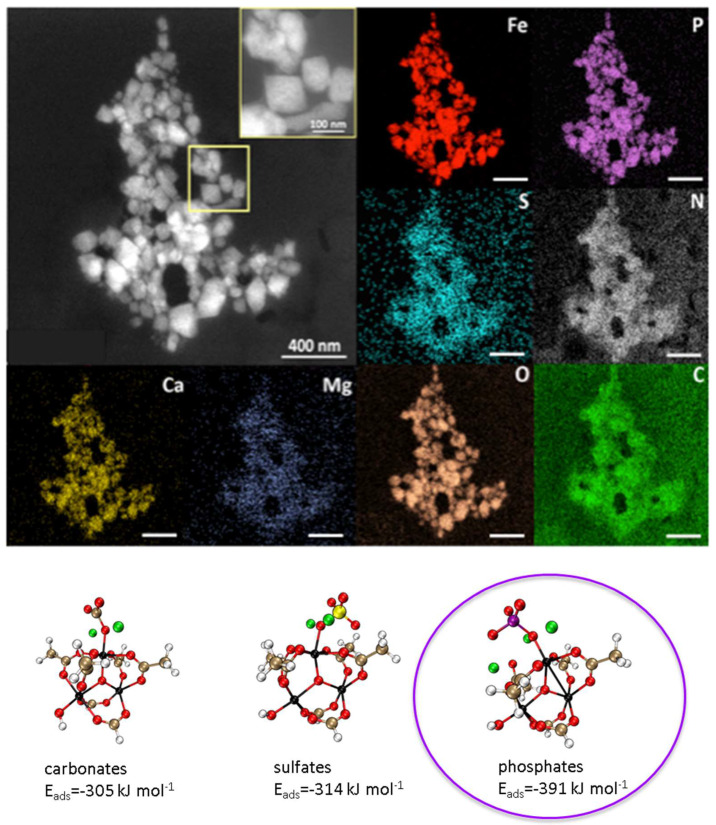
**Upper panel**: STEM-HAADF of nanoMOFs after 48 h incubation in blood. Elemental mapping of blood components into the nanoMOFs. Fe (red), P (violet), S (blue), N (white), Ca (yellow), Mg (dark blue), O (brown), and C (green). **Lower panel**: DFT-calculated interaction energies of ions@Fe-CUS for sodium carbonate, sulfate, and phosphate. Color code: C (brown), O (red), H (white), ion (black), S (yellow), P (purple), and Na (green). Scale bar represents 400 nm, unless stated.

The nanoMOFs’ degradation could be attributed to the presence of various ions and other molecules from blood and plasma. Indeed, it was found that the nanoMOFs were enriched in various elements, mainly N, P, S, Ca, and Mg (Figure 9), which were not initially in the nanoMOFs. Other elements were in trace amounts. We speculate that N could be essentially attributed to proteins, which were demonstrated to bind onto the nanoMOFs readily [46,47].

As P and N were the main elements in the composition of the nanoMOFs in contact with blood or serum, the P/Fe and N/Fe ratios were calculated for different regions of interest (single nanoMOF or groups of particles), as previously described. P was evenly distributed in both nanoMOF after contact with serum and blood, with a constant ratio of 0.9 ± 0.1 and 0.5 ± 0.1, respectively, after early (2 h) incubation (Figure S6, Tables S4 and S5). Noteworthy, despite the significantly higher concentration of HCO_3_^−^ (27 mmol L^−1^), Ca^+2^ (2.5 mmol L^−1^), and Mg^+2^ (1.5 mmol L^−1^) concentrations in the blood compared with HPO_4_^2−^ (only around 1.0 mmol L^−1^), P was largely detected in the nanoMOFs’ structures [^44^]. As mentioned before, C atoms may refer to the carboxylate ligand, the samples’ holder, and the HCO^3−^ from the blood. Thus, XEDS analysis hampers the distinction between different C elements. However, Ca and Mg were found in traces, and the calculated ratios could not be precisely defined within reason of the detection limits. Figure 9 shows the elementary mapping of each element present in the nanoMOFs’ structures upon their degradation in blood after 48 h. These findings suggest a higher affinity of P-based compounds for the nanoMOFs as compared with other ones and are in line with the trend obtained for the DFT calculations of the interaction energies between CO_3_^−^, SO_4_^2−^ and HPO_4_^2−^ and the oxo-trimer cluster described in Figure 8, with the higher interaction energies being obtained for HPO_4_^2−^.

As stated previously, the nanoMOF enrichment with N could be attributed to their interaction with the large variety of proteins present in the two studied media, especially albumin. Indeed, the N/Fe ratio, representative of protein/nanoMOF binding, was found at 1.1 ± 0.5 (serum 2 h) (Table S4) and 1.9 ± 0.8 (blood 2 h) (Table S5).

In contrast to the homogeneous distribution of P in the nanoMOFs, N was not evenly presented, as shown by the large standard deviations (0.4 and 0.9 for N as compared with 0.1 for P). More specifically, the N distribution was strongly dependent on the nanoMOFs’ sizes. Figure S7 is a typical example, showing that the N content was higher on small nanoMOFs as compared with the largest ones. Possibly, proteins remain adsorbed onto the nanoMOFs’ surfaces, which explains why the smallest particles with the highest external surface areas exhibit a larger signal coming from proteins. 

## 3. Materials and Methods

### 3.1. Materials

Iron(III) chloride hexahydrate 98% (Alfa Aesar, Kandel, Germany) and 1,3,5-benzene tricar-boxylic acid, 95% (Sigma-Aldrich, Saint-Quentin-Fallavier, France) were used for the synthesis of nanoMOFs. Absolute ethanol 99% (Thermo Fischer Scientific, Les Ulis, France) was used for the purification of nanoMOFs. Prednisolone (Sigma-Aldrich, Saint-Quentin-Fallavier, France), prednisolone phosphate (sodium salt) (Cayman Chemical, Michigan, USA), and methyl prednisolone sulfate (triethylamine salt) (Clearsynth, Mumbai, Maharashtra, India) were the active molecules studied for drug encapsulation. Acetonitrile and methanol (HPLC grade) were purchased from Sigma-Aldrich (Saint-Quentin-Fallavier, France) as organic mobile phases for the detection of AIs and ligands. For the preparation of aqueous buffer solutions for HPLC measurements, ammonium acetate 99% (Alfa Aesar, Kandel, Germany), NaH_2_PO_4_, and Na_2_HPO_4_ (Sigma-Aldrich, Saint-Quentin-Fallavier, France) were used. Ortho-phosphoric acid (≥85%) and potassium hydroxide (Sigma-Aldrich, Saint-Quentin-Fallavier, France) were used for the adjustment of the pH. DPBS (1X, pH = 7.4) was purchased from Thermo Fischer Scientific (Les Ulis, France); it contains 1.47 mM KH_2_PO_4_, 8.59 Na_2_HPO_4_.7H_2_O, 137 mM NaCl, and 2.66 mM KCl. Anhydrous sodium sulfate (99%, abcr, GmhB, Karlsruhe, Germany) and sodium phosphate dibasic (Sigma-Aldrich, Saint-Quentin-Fallavier, France) were purchased for release studies. Serum and blood were recov-ered from BALB/c mice after their sacrifice. Milli-Q water was obtained from a Millipore apparatus equipped with a 0.22μm filter (18.2 MΩ cm). Reagents and solvents were used without further purification.

### 3.2. Synthesis of nanoMOFs

NanoMOFs were prepared by microwave-assisted hydrothermal synthesis from a mixture of iron chloride hexahydrate (8.97  mmol) and 1,3,5-benzene tricarboxylic acid (4.02  mmol) in 20 mL of deionized water, as previously described [7,18]. The mixture was heated for 6 min at 130 °C under stirring (ramp time: 1.30 min, hold time: 4.30 min). The power applied was 800 Watts (Mars-5, CEM, USA) with a maximum power output and pressure of 800 Watts. At the end of the heating procedure, the reaction vessel with the same quantity of water (20 mL) was added to the mixture, and it was placed in an ice bath for 10 min to stop the nucleation process. The synthesized nanoMOFs were recovered by centrifugation (14,000× *g*) and washed with absolute ethanol 6 times to remove the residual non-reacted trimesate. Lastly, centrifugation was performed (4000× *g*, 1 min) to separate the smaller nanoMOFs from the larger nanoMOFs. The final product was stored in ethanol until final use, and the particles were either dried or re-suspended in aqueous media, depending on the characterization technique.

### 3.3. Characterization of nanoMOFs

A series of characterization studies were used to check the successful synthesis of MIL-100(Fe) nanoMOFs. Their morphology was observed by TEM/STEM studies performed on a Titan Themis microscope corrected for spherical aberrations on the probe. The microscope was equipped with the “Super-X” analysis XEDS system with 4 SDD detectors allowing it to achieve a solid angle of 0.8 steradians.

The observations were made at 200 kV with a sufficiently low probe current, i.e., around 40 to 50 pA, so as not to degrade the sample. The STEM-HAADF–XEDS chemical maps were carried out under the same conditions for an acquisition time of approximately 15 to 20 min.

For the HAADF-STEM images acquisition, the half-angle of convergence of the probe was 17 mrad, and the collection half-angle of the Annular Dark Field detector was 69 mrad (inner angle) and 200 mrad (outer angle).

For the TEM grid preparation, a 2 μL drop of the solution was placed on a 200-mesh copper grid covered with a pure carbon membrane (from Ted Pella).

Mean hydrodynamic diameter and size distribution were obtained by Dynamic Light Scattering (DLS, Malvern Nano ZS, Zetasizer Nano series, France). Surface charge was evaluated by measuring the ζ-potential with a Zetasizer NanoZS. Samples were prepared to a final concentration of 0.1 mg mL^−1^.

The crystallinity of nanoMOFs was recorded on a D8 Advance Bruker Diffractometer in Debye–Scherrer geometry in the 2θ range of 2–40°. The diffractometer was equipped with a Ge (111) monochromator producing Cu Kα_1_ radiation (λ = 1.540598 Å) and a LynxEye detector.

Infrared spectra showing the chemical composition of the nanoparticles were evaluated with a Nicolet iS5 FT-IR ThermoFisher spectrometer between 400 and 4000 cm^−1^.

### 3.4. Drug Encapsulation

Drug loading was performed via a green procedure by simple impregnations of nanoMOFs into aqueous drug solutions. Before that, nanoMOFs ethanolic suspensions (0.5 mg mL^−1^) were extensively washed with Milli-Q water. Continuously, they were re-dispersed in aqueous drug solutions (0.15 mg mL^−1^). All the suspensions were gently mixed overnight at RT. Afterward, they were centrifuged (17,000× *g*, 20 min), and the supernatants were analyzed by HPLC to quantify the amount of non-encapsulated drug.

The theoretical loading (TL) was defined using the equation:

TL (%) = (Drug solution (mg))/(nanoMOFs (mg)) × 100.(1)

The drug loading (DL) and the encapsulation efficiency (EE) were calculated using the following equations:

DL (%) = (Encapsulated drug (mg))/(nanoMOFs (mg)) × 100(2)

and

EE (%) = (Encapsulated drug (mg))/(initial drug (mg)) × 100(3)

where the “encapsulated drug” represents the amount (mg) of the entrapped drug in nanoMOFs, and the “initial drug” represents the amount (mg) of total drug available in the starting solution.

### 3.5. Drug and Ligand Release

The loaded pellets (0.5 mg) were re-suspended in 1 mL of each solution and incubated at 37 °C under continuous stirring to compare release studies in different media. At different time points (0 h, 0.5 h, 1.5 h, 3 h, 6 h, 24 h, 48 h, and 72 h), the supernatants were recovered by centrifugation (17,000× *g*, 20 min) and analyzed by HPLC to quantify the released drug and the corresponding trimesate ligand.

### 3.6. HPLC Measurement Conditions

Drug content and release, as well as the release kinetics of the corresponding trimesate, was followed by high-performance liquid chromatography (HPLC).

Different conditions were used for the quantification of the drugs and trimesic acid by HPLC.

#### 3.6.1. Quantification of Prednisolone

The detection of prednisolone was carried out in a reversed-phase HPLC system (Agilent 1100, USA) equipped with a Phenomenex C18 column (4.6 mm × 250 mm, 5 µm). 

Prednisolone was analyzed according to the official European Pharmacopoeia Method [48]. A flow rate of 1 mL min^−1^ was used. The temperature was fixed at 40 °C, and the injection volume was 10 μL. The mobile phase contained A (water): B (MeCN) (50:50 *v/v*), and the drug was detected through a gradient program consisting of the following steps.

40% of B was held for 14 min, then B was gradually increased to 80% until 20 min were reached, and finally, it was kept constant for 5 more minutes. The sample was monitored by UV absorbance at 254 nm. The retention time of prednisolone was 8.7 min.

#### 3.6.2. Quantification of PP

The detection of PP was carried out in a reversed-phase HPLC system, Waters Alliance e2695 Separations Module (Waters, Milford, MA, USA), equipped with a UV-Vis detector Waters 2998. The system was controlled by Agilent software. SunFire-C18 reverse-phase column (5 μm, 4.6 mm × 150 mm, Waters) was employed, and the analysis method was adjusted as previously reported [49]. More precisely, an isocratic method with a mobile phase containing A (acetate buffer 0.01 M, pH = 7), B: ACN: C: MeOH (75:20:5 *v/v/v*) at a flow rate of 0.8 mL min^−1^ was used. The temperature was fixed at 30 °C, and the injection volume was 20 μL. The sample was monitored by UV absorbance at 254 nm. The retention time of prednisolone sodium phosphate was 6.3 min.

#### 3.6.3. Quantification of PS

The detection of PS was carried out in a reversed-phase HPLC system (Agilent 1100, USA) equipped with a Phenomenex C18 column (4.6 mm × 250 mm, 5 µm). The system was controlled by Agilent software. A flow rate of 1 mL min^−1^ was used. The temperature was fixed at 25 °C, and the injection volume was 50 μL. The mobile phase consisted of A: ammonium acetate buffer (0.01 M): B (ACN) (90:10), and the drug was detected through a gradient program consisting of the following steps.

For the first 10 min, 10% of B was gradually increased to 90%, then it was kept constant for 15 more minutes, and finally, it was increased back to 10% for the last 5 min. The sample was monitored by UV absorbance at 286 nm. The retention time of methylprednisolone sulfate was 7.9 min.

#### 3.6.4. Quantification of Trimesate Ligand Release

The detection of trimesic acid was carried out in a reversed-phase HPLC system, Waters Alliance e2695 Separations Module (Waters, Milford, MA, USA), equipped with a UV-Vis detector Waters 2998. The system was controlled by Agilent software. SunFire-C18 reverse-phase column (5 μm, 4.6 mm × 150 mm, Waters) was employed. For the analysis of BTC, a mobile phase, A, consisting of a buffer solution (0.04 M, pH = 2.5), and a mobile phase, B, with MeCN (50:50) was used. The injection volume was 50 µL, and the detection wavelength was set at 225 nm. The column temperature was fixed at 25 °C [40]. The retention time of BTC was 3.1 min.

Buffer preparation: NaH_2_PO_4_ (2.4 g, 0.02 mol) and Na_2_HPO_4_ (2.84 g, 0.02 mol) were dissolved in 1 L of Milli-Q water. The pH was then adjusted to 2.5 with H_3_PO_4_ (≥ 85%).

### 3.7. Computational Section

Density Functional Theory calculations: The interactions of prednisolone, PP, and PS with the Fe-CUS present in MIL-100(Fe) were computationally assessed using cluster-based Density Functional Theory (DFT) calculations; The geometry optimization of each drug@Fe-CUS model was performed using B3LYP exchange–correlation functional, as implemented in the Gaussian 09 program suite. The TZVP basis set was employed for all atoms [50]. The DFT-D3 method was implemented to include the dispersion contribution [51]. Several starting configurations for the drugs were considered to converge towards the lowest energy minima. The drug/host interaction energy for all these systems was further calculated by the difference between the energy of the optimized drug@Fe-CUS model and those of the individual constituents, i.e., prednisolone, PP and PS, as well as sodium carbonate, sulfate, and phosphate.

Monte Carlo simulations: the Monte Carlo simulations were carried out to explore the distribution of the prednisolone uptake within the MIL-100(Fe). All atoms of the MOF framework were treated as charged Lennard-Jones (LJ) interacting sites with LJ parameters taken from the Universal force field (UFF) [52] and the DREIDING [53] force field for the inorganic and the organic nodes, respectively. The atomic charges were calculated using cluster Density Functional Theory (DFT) simulations and the electrostatic potential (ESP) method. Regarding the molecule, the general Amber force field (GAFF) [54] atom types with ESP [55] charges were used. The electrostatic potential using the B3LYP functional with the 6-31 + G(d) basis set was employed to extract the partial charges for prednisolone using the CHELPG scheme [56]. The corresponding LJ cross-parameters were obtained using the Lorentz–Berthelot combination. The Ewald summation method was used to calculate the electrostatic interactions, and the treatment of the short-range interactions was considered with a cut-off distance of 12 Å.

## 4. Conclusions

The iron-based MIL-100(Fe) nanoMOFs were shown to act as efficient host matrices, soaking PP from its aqueous solution with high efficiencies until saturation of the iron-available sites. In contrast, the pristine hydrophobic drug prednisolone and PS were loaded with lower efficiencies and were released in a burst manner, highlighting their lower affinity for the nanoMOFs, in agreement with the cluster-based DFT calculations. In addition, only PP could (partially) stabilize the nanoMOFs towards degradation in PBS. It was revealed that, despite massively losing their organic constitutive ligands, the nanoMOFs maintained a faceted structure and similar size before and after degradation. Noticeably, nanoMOF morphology was practically unaltered after 2 days of incubation in biological fluids (blood and serum) despite the appearance of holes at their external surface. The nanoMOFs’ composition, however, drastically changed as a function of the degradation media. STEM-HAADF, in conjunction with XEDS, was a powerful tool to unravel not only drug distribution within the nanoMOFs’ porosity but also changes in their composition upon degradation. Proteins bulkier than the nanoMOFs’ windows were found to be adsorbed at the nanoMOFs’ external surfaces, whereas ions from the suspension media diffused through the nanoMOFs’ porosity. These studies suggest that nanoMOFs interact with both phosphates and proteins in the blood and that these interactions are fast. Iron trimesate nanoMOFs are peculiar in the sense that they can maintain their global morphology despite dramatic changes in their composition according to the medium they are in contact with.

These investigations can be of interest for the design of nanoMOFs as drug carriers and in other fields, such as (bio)catalysis and gas storage. This study paves the way for a deeper understanding of the role of the different moieties, on the drug or in the media, on the stabilization of the framework, and, therefore, on the release kinetics of the drugs and the MOFs’ constitutive ligands. In addition, it opens new possibilities for the treatment of severe diseases using nanoMOFs loaded with prednisolone derivatives through, for example, ocular administration. 

## Data Availability

Data available upon request.

## Supplementary Materials

The supporting information can be downloaded at: https://www.mdpi.com/article/10.3390/ijms24043362/s1. Reference [57] is cited in the supplementary materials.
