# Supplementary material for: Nanoscale Iron-Based Metal–Organic Frameworks: Incorporation of Functionalized Drugs and Degradation in Biological Media
Source: Int J Mol Sci. 2023 Feb 8;24(4):3362. doi: 10.3390/ijms24043362 (PMC9965190; doi:10.3390/ijms24043362)
Supplement: Supplementary file 1 [file ijms-24-03362-s001.zip › ijms-2186560-supplementary.pdf]

Supporting information

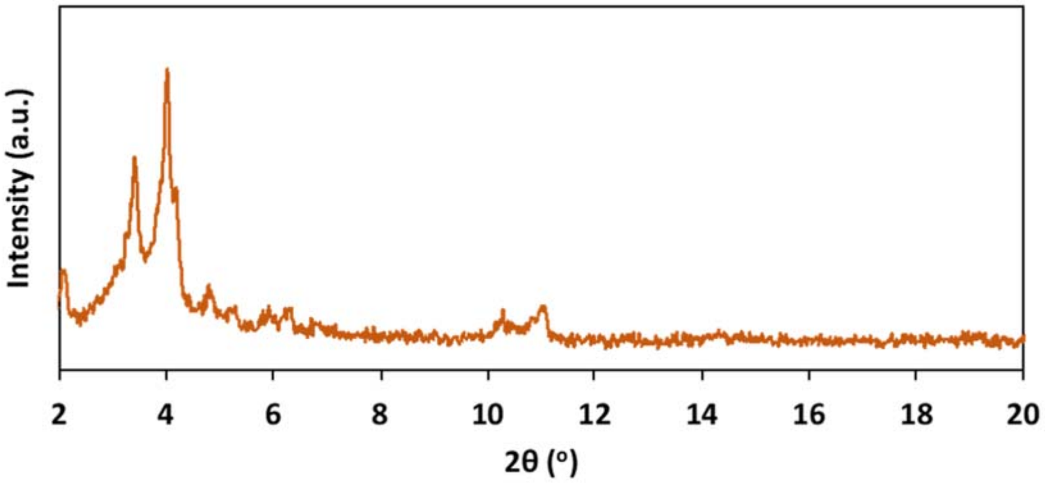

Figure S1: Normalized PXRD patterns of nanoMOFs.

Table S1: Dimensions and volume occupancies of Prednisolone, PP and PS calculated by DFT modeling. As an example, a, b and c sides of Prednisolone are illustrated.

Active Molecule	Volume (Å^3)	Diameter (Å)	Lengths along the 3 sides (Å)		
			a	b	c
Prednisolone	356.6	14.47	14.24	8.51	7.50
PSS	457.6	18.02	17.82	9.53	7.25
PSP	473.8	17.37	17.25	8.38	8.00

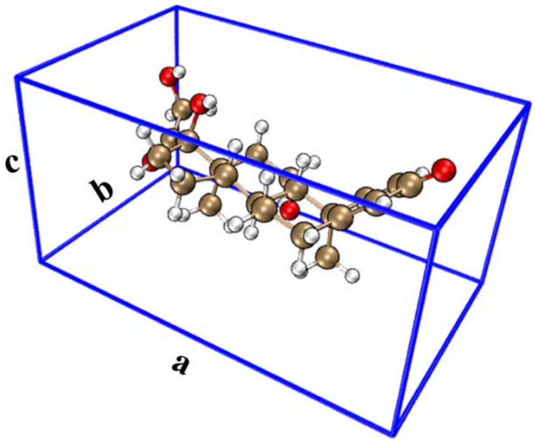

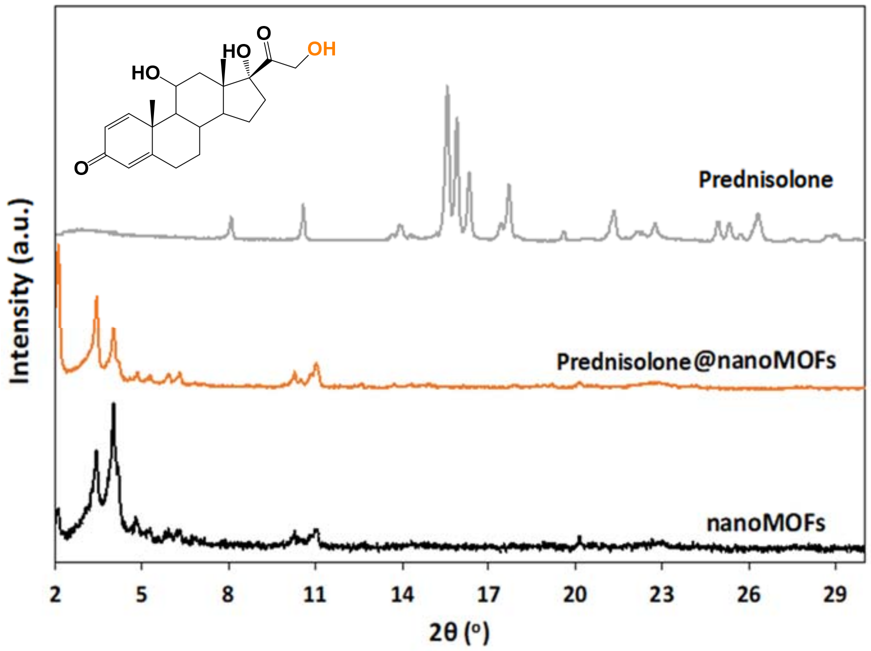
HO
HO
O
OH
O
Prednisolone
Prednisolone@nanoMOFs
nanoMOFs
Intensity (a.u.)
2 5 8 11 14 17 20 23 26 29
2θ (º)

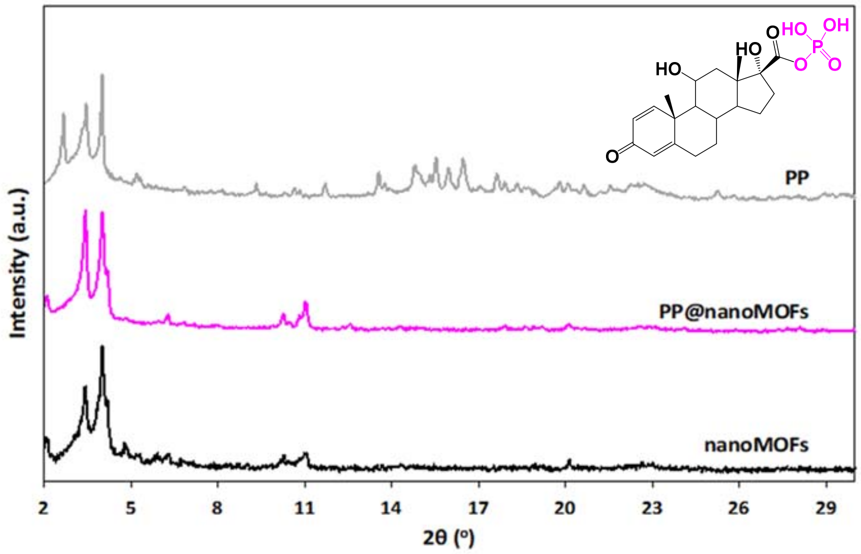
HO
HO
O
HO
OH
P
O
O
O
PP
PP@nanoMOFs
nanoMOFs
Intensity (a.u.)
2 5 8 11 14 17 20 23 26 29
2θ (º)

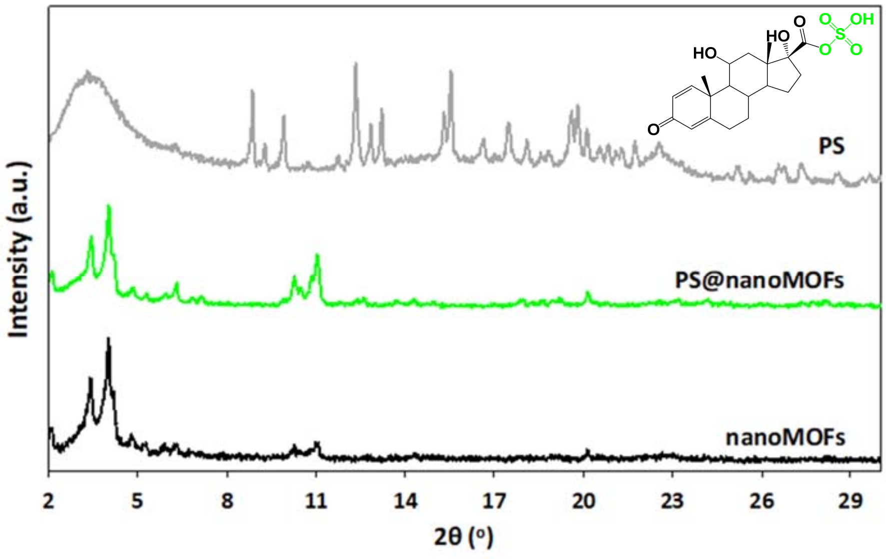
HO
HO
O
O
OH
S
O
O
O
PS
PS@nanoMOFs
nanoMOFs
Intensity (a.u.)
2 5 8 11 14 17 20 23 26 29
2θ (º)

Figure S2: Normalized PXRD patterns of MIL-100(Fe) nanoMOFs before (black) and after encapsulation of Prednisolone (orange), PP (pink) and PS (green).

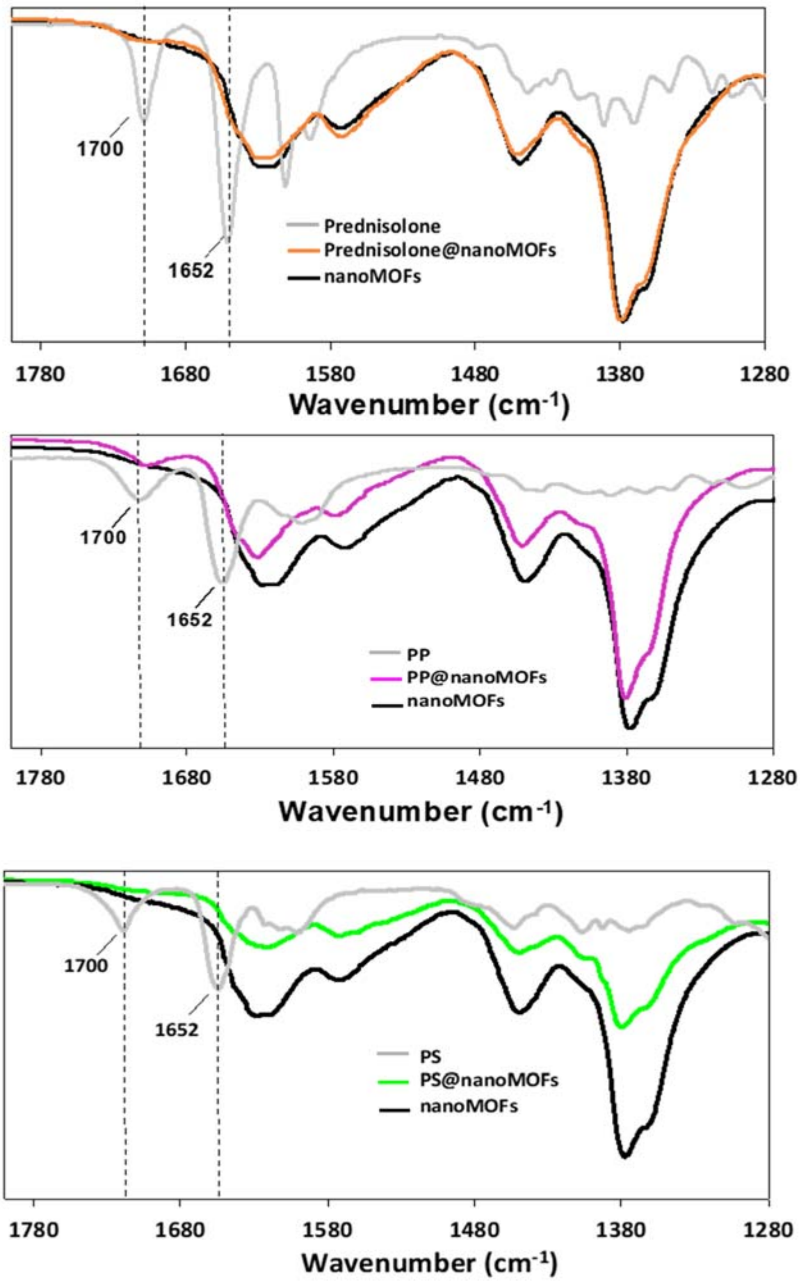

Figure S3: FT-IR spectra of MIL-100(Fe) nanoMOFs before (black) and after drug encapsulation of P (orange), PP (pink) and PS (green). Spectra of pure Prednisolone, PP and PS are presented in grey colors.

Table S2. Elemental distribution (by normalized at.%) and the calculated ratio of P (drug molecule) per each iron trimer. The calculated values correspond to the selected regions of interest highlighted and numbered in Figure 5.

**Regions**	**Fe [normalized at. %]**	**P [normalized at. %]**	**Ratio trimer=3*(P/Fe)**
**1**	**2.0 ± 0.3**	**0.3 ± 0.1**	**0.5**
2	2.8 ± 0.4	0.4 ± 0.0	0.4
3	2.7 ± 0.4	0.3 ± 0.1	0.3
4	2.8 ± 0.4	0.3 ± 0.1	0.3
5	2.3 ± 0.4	0.2 ± 0.1	0.5
6	2.5 ± 0.5	0.2 ± 0.1	0.2
7	2.5 ± 0.4	0.4 ± 0.1	0.5
8	3.8 ± 0.6	0.6 ± 0.1	0.5
9	2.6 ± 0.4	0.4 ± 0.1	0.5
10	2.7 ± 0.5	0.4 ± 0.1	0.4
11	2.5 ± 0.5	0.4 ± 0.1	0.5
12	2.6 ± 0.5	0.5 ± 0.1	0.6
13	2.5 ± 0.5	0.4 ± 0.1	0.5
14	2.4 ± 0.5	0.3 ± 0.1	0.4
15	2.4 ± 0.9	0.4 ± 0.2	0.5

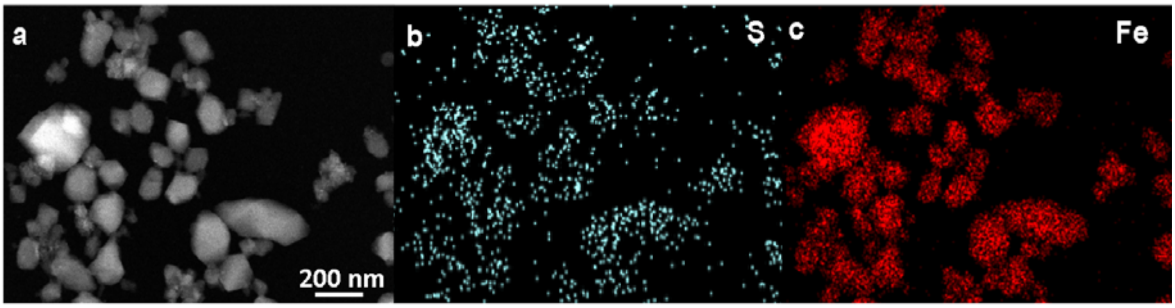

Figure S4. (**a**) Typical STEM-HAADF images of nanoMOFs loaded with PS. (**b**),(**c**) Elemental distribution of Fe (red) and S (blue) into the nanoMOFs after PS loading.

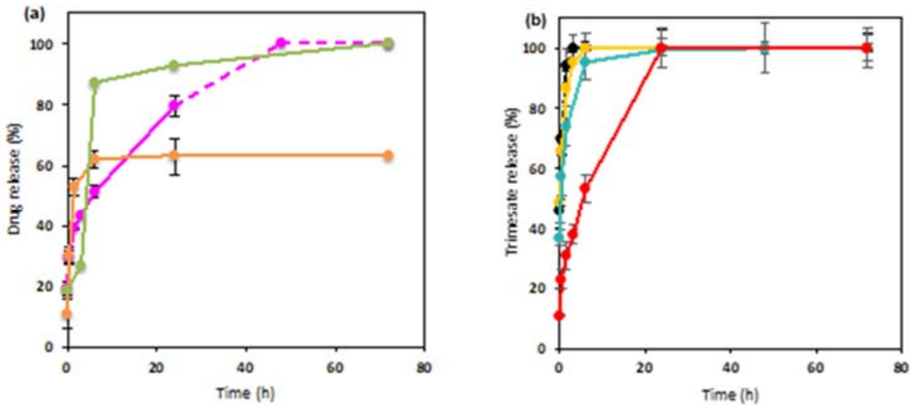

Figure S5. (**a**) Comparison of Prednisolone (orange), PP (pink) and PS (green) release in PBS. b) Trimesate loss of loaded nanoMOFs with P (blue), PS (yellow) and PP (red) and empty nanoMOFs (black). Sample concentration was 0.5 mg mL^{-1}.

Table S3: Solubility of prednisolone in different solvents at 25 °C.

Solvent	Solubility (mg mL^{-1}) at 25 °C
H_2O	0.22
EtOH	33.33
Chloroform	5.55
Acetone	20
MeOH	soluble
Dioxane	soluble

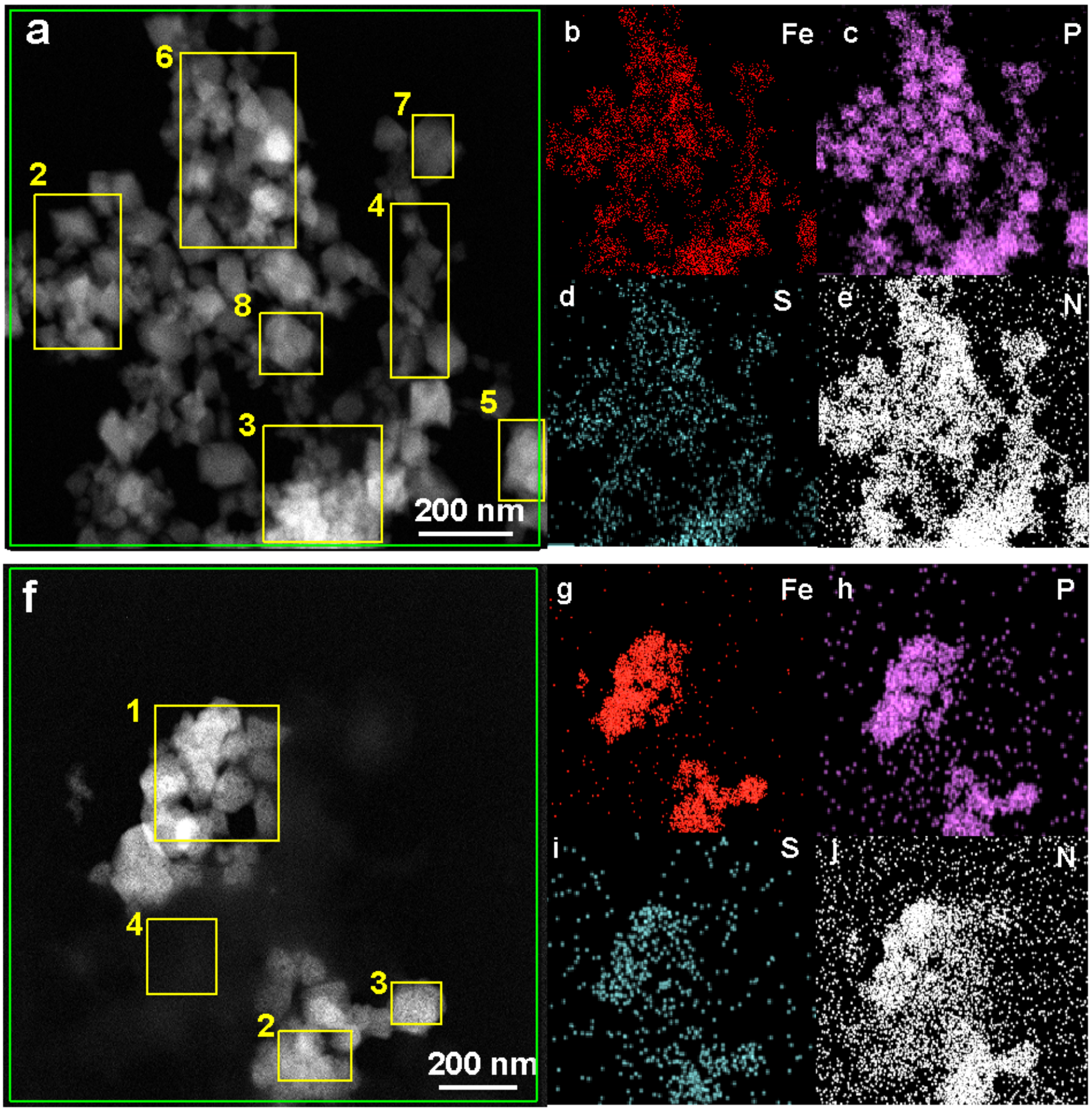

Figure S6. (**a**),(**f**) STEM-HAADF images of nanoMOFs upon incubation in serum (2 h) and in blood (2 h). (**b**),(**c**),(**d**),(**e**),(**g**),(**h**),(**i**),(**j**) Elemental distribution of Fe (red), P (violet), S (blue) and N (white). Yellow rectangles represent the selected regions by STEM-XEDS and green rectangle the entire image.

Table S4: Element distribution (by normalized at. %) and the calculated ratio of N (protein) per Fe (nanoMOFs) after 2 h incubation in serum (0.5 mg mL $^{-1}$). The calculated values correspond to the selected regions of interest as numbered in microscopic images (**a**) and (**b**). Region numbered 1 represents the average values (represented as green rectangles).

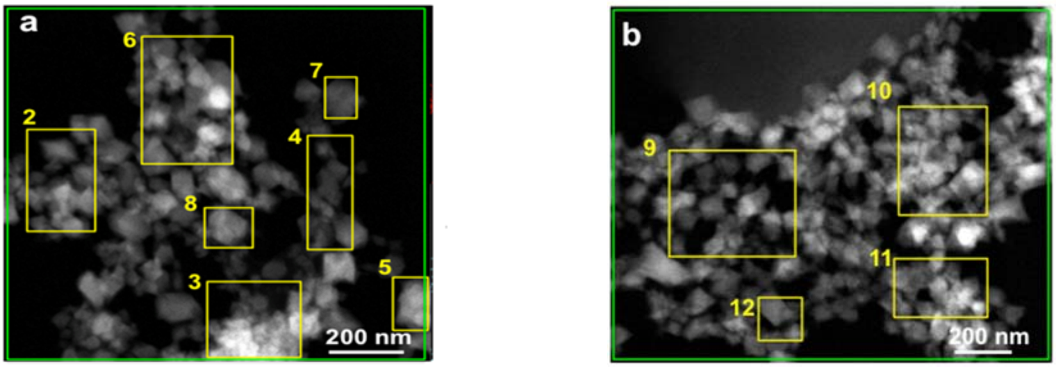

Regions	Fe [normalized at. %]	N [normalized at. %]	P [normalized at. %]	Ratio trimer=N/Fe	Ratio trimer=P/Fe
**1**	**4.0 ± 1.1**	**4.1 ± 1.6**	**3.4 ± 0.9**	**1.1 ± 0.5**	**0.8 ± 0.1**
2	3.6 ± 0.5	3.8 ± 0.2	3.0 ± 0.2	1.1	0.8
3	4.1 ± 0.5	6.9 ± 0.1	3.3 ± 0.1	1.7	0.8
4	2.6 ± 0.5	3.5 ± 0.2	1.9 ± 0.2	1.4	0.7
5	4.7 ± 0.7	2.7 ± 0.2	4.3 ± 0.3	0.6	0.9
6	2.8 ± 0.3	4.0 ± 0.1	3.0 ± 0.1	1.4	0.8
7	3.8 ± 0.9	2.8 ± 0.4	3.3 ± 0.5	0.7	0.9
8	4.3 ± 1.0	2.0 ± 0.3	3.9 ± 0.6	0.5	0.9
9	3.5 ± 0.5	5.1 ± 0.1	2.5 ± 0.1	1.5	0.7
10	4.3 ± 0.5	4.9 ± 0.1	3.6 ± 0.1	1.0	0.8
11	3.9 ± 0.5	6.3 ± 0.2	3.1 ± 0.1	1.6	0.8
12	6.7 ± 0.1	2.9 ± 0.3	5.3 ± 0.8	0.4	0.8

Table S5: Element distribution (by normalized at. %) and the calculated ratio of N (protein) per Fe (nanoMOFs) after 2h incubation in blood (0.5 mg mL $^{-1}$). The calculated values correspond to the selected regions of interest as numbered in microscopic images (**a**) and (**b**). Region numbered 1 represents the average values (represented as green rectangles). Scale bar represents 200 nm.

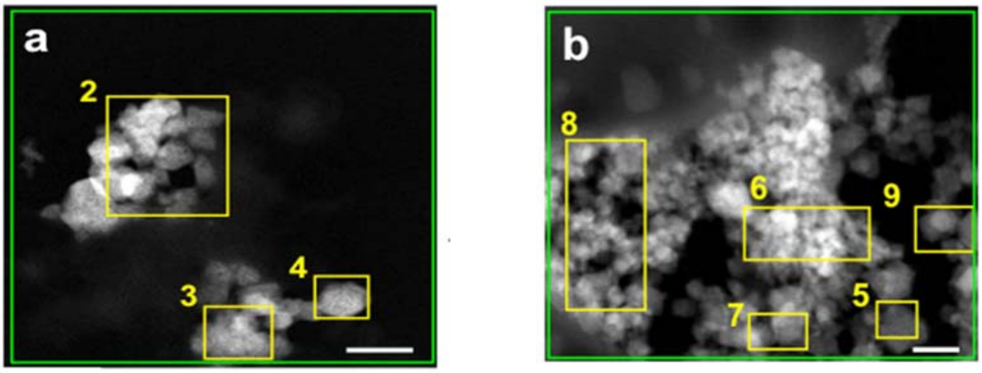

Regions	Fe [normalized at. %]	N [normalized at. %]	P [normalized at. %]	Ratio trimer=N/Fe	Ratio trimer=P/Fe
**1**	**3.0 ± 0.6**	**5.2 ± 0.2**	**1.6 ± 0.5**	**1.9 ± 0.8**	**0.5 ± 0.1**
2	2.7 ± 0.4	5.1 ± 0.2	1.5 ± 0.1	1.9	0.5
3	1.9 ± 0.4	7.0 ± 0.3	0.9 ± 0.2	3.7	0.5
4	3.3 ± 0.8	4.2 ± 0.4	1.8 ± 0.3	1.3	0.5
5	4.1 ± 0.6	3.4 ± 0.2	2.6 ± 0.3	0.8	0.6
6	3.2 ± 0.4	6.1 ± 0.1	1.5 ± 0.3	1.9	0.5
7	2.7 ± 0.4	5.7 ± 0.1	1.3 ± 0.1	2.1	0.5
8	3.1 ± 0.4	5.5 ± 0.1	1.6 ± 0.1	1.8	0.5
9	2.6 ± 0.4	4.7 ± 0.2	1.5 ± 0.2	1.8	0.6

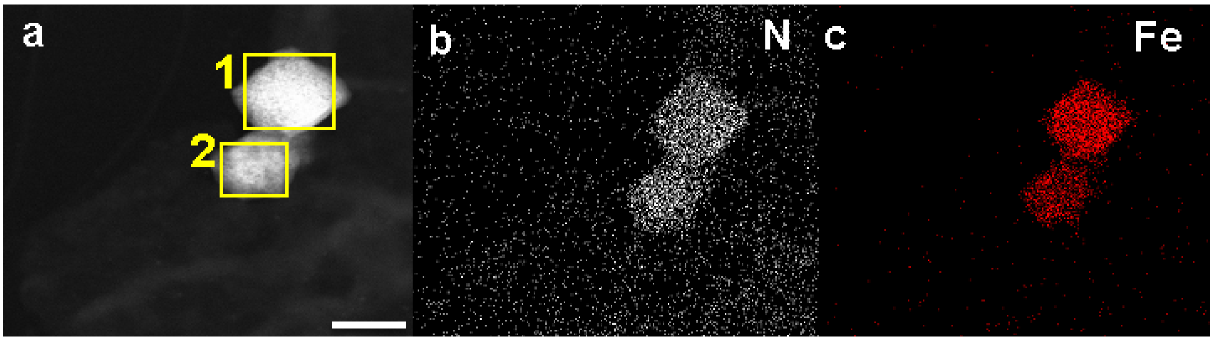

Figure S7: (**a**) STEM-HAADF image of nanoMOFs after 2 h incubation in blood. (**b**), (**c**) Elemental distribution of Fe (red) and N (white). The calculated N/Fe was found 1.4 for region 1 and 3.5 for region 2, suggesting that protein is prone to get adsorbed on the external surface of smaller nanoMOFs. Scale represents 200 nm.

Theoretical calculations

The MTN framework consists of two different cage systems, namely [5^{12}] and [$5^{12}6^{4}$] [1].

The small [5^{12}] cages are interconnected and accessible only through narrow openings of around 4.8-5.8 Å which makes them inaccessible to the drugs Prednisolone, PP and PS studied here. All the large [$5^{12}6^{4}$] cages are interconnected through their hexagonal windows (openings around 8.6 Å), so the drugs can penetrate through and locate inside the large cages (Table S1).

According to the MTN secondary building units (SBUs) structure, the number of small cages is twice that of the large cages [2].

Formula of nanoMOFs (MW) made of iron trimesate: $Fe_3O(H_2O)_2OH(BTC)_2$ x nH_2O

where nH_20 represents the bound water molecules, which are not taken into account in these calculation as drug loading is expressed with regard to the dried MOF material.

MW = 210 x 2 + 56 x 3 + 16 x 4 + 5 = 657 g/mol, so the total available iron site concentration is:
2/ 657 = 3.0 mmol/g (because 2 coordinated water molecules can be replaced by the drug)

Large cages consist of 28 super tetrahedra and 20 small ones.

Given the fact there are twice as more small cages than large ones, the % of iron trimers accessible to large molecules such as PP is:

% =28 / (2x20+28) = 41.17

So the available sites in large cages are: 3.0 x 0.4117 = 1.235 mmol/g

A loading of 30% (0.3 g/g) of the PP (Mw=484 g/mol) in nanoMOFs corresponds to:

0.3 (g/g)/ 484 (g /mol) = 0.619 mmol/g of MOF

References

1. Lin, Z. S.; Chen, D.; Nie, H. Y.; Wong, Y. T. A.; Huang, Y. Investigations of the Formation of Zeolite ZSM-39 (MTN). *Can. J. Chem.* **2019**, *97*, 840–847.
2. Agostoni, V.; Chalati, T.; Horcajada, P.; Willaime, H.; Anand, R.; Semiramoth, N.; Baati, T.; Hall, S.; Maurin, G.; Chacun, H.; Bouchemal, K.; Martineau, C.; Taulelle, F.; Couvreur, P.; Rogez-Kreuz, C.; Clayette, P.; Monti, S.; Serre, C.; Gref, R. Towards an Improved Anti-HIV Activity of NRTI via Metal-Organic Frameworks Nanoparticles. *Adv. Healthc. Mater.* **2013**, *2*, 1630–1637.
